# Thiamine Deficiency in a Patient With Schizophrenia: Precautions and Countermeasures for Subclinical Thiamine Deficiency

**DOI:** 10.7759/cureus.38454

**Published:** 2023-05-02

**Authors:** Mayumi Ishida, Nozomu Uchida, Akira Yoshioka, Izumi Sato, Hiroshi Ito, Ryota Sato, Naoki Mizunuma, Hideki Onishi

**Affiliations:** 1 Department of Psycho-oncology, Saitama Medical University International Medical Center, Hidaka, JPN; 2 Department of General Medicine, Ogano Town Central Hospital, Ogano, JPN; 3 Department of Supportive Medicine, Saitama Medical University International Medical Center, Hidaka, JPN; 4 Department of Clinical Oncology, Mitsubishi Kyoto Hospital, Kyoto, JPN; 5 Department of Clinical Epidemiology, Nagasaki University, Graduate School of Biomedical Sciences, Nagasaki, JPN; 6 Department of General Medicine, Ito Internal Medicine and Pediatric Clinic, Fukuoka, JPN; 7 Department of Pharmacy, Maruki Memorial Medical and Social Welfare Center, Moroyama, JPN

**Keywords:** thiamine deficinecy, korsakoff’s syndrome, schizophrenia, non-alcoholic wernicke's encephalopathy, thiamine

## Abstract

Patients with schizophrenia often experience problems associated with ordinary exercises of life due to their mental symptoms. Those experiencing problems related to feeding behavior, in particular, are considered to be susceptible to developing Wernicke encephalopathy due to a deficiency in thiamine, the physiological stores of which are limited; however, there are few reported cases, and most of them were accompanied by the classical triad of signs. We report our experience with asymptomatic thiamine deficiency (TD) in a schizophrenia patient. A 73-year-old female was receiving medication for schizophrenia as an outpatient. No symptoms such as hallucinations or delusions were observed, the patient had a sociable personality and was able to function at a level where she could live alone. Although there were no active complaints about eating by the patient, we investigated the situation due to reports of TD in schizophrenia patients. As results revealed a significant decrease in whole blood thiamine to 19 ng/mL (reference range: 24-66 ng/mL), we administered a large dose of thiamine. No changes were observed in psychosomatic symptoms before and after administration. Patients with schizophrenia experience problems that may lead to TD, such as dietary imbalances and disturbed feeding habits. Therefore, even if patients with schizophrenia do not actively complain about their feeding behavior, it may be necessary to take medical measures such as blood sampling in consideration of the potential for developing TD.

## Introduction

Thiamine, in its biologically active form thiamine pyrophosphate, is an essential coenzyme for oxidative cellular metabolism. However, as thiamine cannot be synthesized in vivo and its physiological store is limited, thiamine deficiency (TD) can occur after 2-3 weeks with a reduced appetite [1].

Wernicke encephalopathy (WE) is a neuropsychiatric disorder that is caused by TD [2]. A typical case presents with a triad of signs consisting of altered mental state, cerebellar ataxia, and ophthalmoplegia, but these symptoms are not disease-specific, and only 16% of patients develop all 3 signs, with 19% of patients being asymptomatic [3, 4].

Schizophrenia has a lifetime prevalence of close to 1% [5], making its treatment and care a major public health challenge. Patients with schizophrenia display certain characteristics that make them prone to TD and WE, such as inadequate self-care, poor feeding habits, and malnutrition [6]. However, a systematic review revealed that only 15 patients with schizophrenia were diagnosed with WE, and 80% of the reported cases presented with the classical triad [7]. Given the prevalence of the classical triad of signs was 16% [3], it is possible that WE and TD are overlooked in patients with schizophrenia. However, at present, research on the potential for schizophrenia possibility to develop TD and WE has not been expanded.

Here, we report our experience of asymptomatic TD in a patient with schizophrenia undergoing outpatient care.

## Case presentation

The patient was a 73-year-old female who had developed delusions, hallucinations, and markedly impaired functioning and was diagnosed with schizophrenia at the age of 48. She had no history of alcohol or drug dependence.

Her first consultation at our department was 5 years previously, and she had not taken psychotropic drugs for 4 years prior to the first consultation. Hallucinations, delusions, and thought broadcasting and markedly impaired functioning were noted at the first consultation, and a recurrent episode of schizophrenia was diagnosed. These symptoms disappeared in 2 weeks with 1mg of risperidone. Since then, she has lived alone without any problems, and she remains sociable and has many friends.

The patient has continued oral treatment while monitoring the side effects of drug administration. However, as there have been reports of cases of WE and TD among schizophrenia patients, and as schizophrenia patients display characteristics in terms of ordinary exercises of life and feeding that can lead to WE and TD, testing for B group vitamins was added to regular blood testing.

Results revealed that the patient’s thiamine level was significantly decreased to 19 ng/mL (reference range: 24-66 ng/mL). Her vitamin B12 was 185 pg/mL (reference range: 180-914 pg/mL) and her folic acid was 8.4 (reference range: ≧4 ng/mL), both of which were within the normal range (Table 1).

**Table 1:** Laboratory examination

Variable	Values	reference range
White Blood Cell count	5.55	3.30-8.60 x10^3^
Red Blood Cell count	4.60	3.58-4.90 x10^6^
Hemoglobin	10.4	11.60-14.80 g/dL
Hematocrit	34.6	35.10-44.40 %
Mean corpuscular volume (MCV)	75.2	83.60-98.20fL
Mean Corpuscular Hemoglobin (MCH)	22.6	27.50-33.20pg
Mean Corpuscular Hemoglobin Concentration (MCHC)	30.1	31.70-35.30g/dL
Platelet	325	158.00-348.00 x10^3^
Albumin	3.9	3.90-4.90 g/dL
creatinine kinase (CK)	61	43.00-165.00 U/L
aspartate aminotransferase (AST)	22	8.00-38.00 U/L
alanine aminotransferase (ALT)	22	4.00-44.00 U/L
lactate dehydrogenase (LDH)	177	106-211 U/L
alkaline phosphatase (ALP)	362	104-338 U/L
γ-glutamil transpeptidase (γ-GTP）	43	0.00-73.00 U/L
Creatinine (Cr)	0.57	0.34-0.79 mg/dL
Sodium (Na)	142	138.00-147.00 mEq/L
Chloride (Cl)	107	98.00-110.00 mEq/L
Potassium (K)	3.9	3.30-4.80 mEq/L
Glucose	124	70-109 mg/dL
vitamin B1	19	24–66 ng/mL
vitamin B12	185	180–914 pg/ml
Folic acid	8.4	≧4 ng/mL

Based on these findings, she was diagnosed with subclinical TD. The patient’s height at the time of blood sampling was 152 cm, and her weight was 60 kg (BMI = 26.0). Neurological examination did not reveal any change in mental status, dizziness, or ocular symptoms. In addition, the subject did not report any imbalance in her diet, decrease in food intake, or change in body weight. The patient had no history of diabetes. Although 200 mg of thiamine was administered intravenously, no change in symptoms was observed between before and after administration. As thiamine deficiency is common in cancer patients, this patient was also screened for cancer and found no abnormalities.

She was also administered 100mg /day of oral thiamine. The patient’s progress has been followed for the two years since, but no significant changes in psychiatric or physical symptoms have been observed. Thiamin has been measured multiple times to confirm no deficiency.

## Discussion

We identified asymptomatic TD in a patient with schizophrenia undergoing drug therapy.

In a study of patients with schizophrenia who developed WE, a high percentage of patients presented with the classical triad; however, as various other psychosomatic symptoms were also observed, it has been pointed out that the detection of WE may be delayed in patients with schizophrenia [7, 8]. In particular, when hallucinations and delusions are observed, these symptoms may be regarded as aggravation of psychiatric symptoms and TD is often overlooked [6].

In the case presented herein, there were no active psychiatric symptoms and no neurological symptoms suggestive of TD. Although TD has been reported in many cancer patients [9], no particular abnormalities were observed on cancer screening. However, given the wide variety of neuropsychiatric symptoms in patients with TD, as well as the presence of asymptomatic cases [4], it is quite possible that some patients with schizophrenia present with subclinical TD and WE without the classical triad of signs, as in the present case.

The reason for suspecting TD in the present case was previous research that revealed TD may occur in schizophrenia patients. Patients with schizophrenia are more likely to develop problems that predispose them to TD, such as psychiatric symptoms, dietary imbalances, and disturbances to feeding behavior, and many of them are unaware of these problems. This is true for the present case, but as the patient lives alone it is difficult to confirm her living conditions other than through her own complaints. Based on such circumstances, it is possible that TD may be overlooked in many similar cases. To prevent this, it is necessary to remain aware of the possibility of TD, perform regular blood sampling, and administer prophylactic thiamine.

The case presented herein has one limitation. Although the patient declared that she did not have any dietary imbalances, as mentioned above, there was no way to confirm her diet with anyone other than the patient herself, so we cannot deny the existence of a dietary imbalance of which the patient was unaware.

## Conclusions

In conclusion, we identified subclinical TD in a patient being treated for schizophrenia and suggest that TD may be overlooked in patients with schizophrenia. Further research will clarify the prevalence of TD in schizophrenia.
